# Codon Optimization Significantly Improves the Expression Level of ***α***-Amylase Gene from *Bacillus licheniformis* in *Pichia pastoris*

**DOI:** 10.1155/2015/248680

**Published:** 2015-06-10

**Authors:** Jian-Rong Wang, Yang-Yuan Li, Dan-Ni Liu, Jing-Shan Liu, Peng Li, Li-Zhi Chen, Shu-De Xu

**Affiliations:** ^1^Guangdong VTR Bio-Tech Co., Ltd., Science and Technology Industry Zone, Nanping, Zhuhai, Guangdong 519060, China; ^2^Guangdong Feed Additive Research and Biotechnology Center, Zhuhai, Guangdong 519060, China

## Abstract

*α*-Amylase as an important industrial enzyme has been widely used in starch processing, detergent, and paper industries. To improve expression efficiency of recombinant *α*-amylase from *Bacillus licheniformis* (*B. licheniformis*), the *α*-amylase gene from *B. licheniformis* was optimized according to the codon usage of *Pichia pastoris* (*P. pastoris*) and expressed in *P. pastoris*. Totally, the codons encoding 305 amino acids were optimized in which a total of 328 nucleotides were changed and the G+C content was increased from 47.6 to 49.2%. The recombinants were cultured in 96-deep-well microplates and screened by a new plate assay method. Compared with the wild-type gene, the optimized gene is expressed at a significantly higher level in *P. pastoris* after methanol induction for 168 h in 5- and 50-L bioreactor with the maximum activity of 8100 and 11000 U/mL, which was 2.31- and 2.62-fold higher than that by wild-type gene. The improved expression level makes the enzyme a good candidate for *α*-amylase production in industrial use.

## 1. Introduction

*α*-Amylases (E.C.3.2.1.1) are classified as a member of family 13 of the glycosyl hydrolases and catalyze the hydrolysis of internal *α*-1,4-O-glycosidic bonds in polysaccharides with the retention of *α*-anomeric configuration in the products [1]. *α*-Amylases are one of the most important industrial enzymes that have a wide variety of applications in starch processing, paper industries, detergent, and so on [2, 3]. *α*-Amylases are ubiquitous enzymes produced by plants, animals, and microorganisms. Although there are many sources of *α*-amylases, microorganisms are the most important sources of *α*-amylases for industrial purposes due to advantages such as less time and space required for production, cost effectiveness, and ease of process modification and optimization [4].

In recent years, many kinds of *α*-amylase have been isolated from various microorganisms, such as bacteria and fungi. Among bacteria,* Bacillus* sp. is widely used for *α*-amylase production to meet industrial needs.* Bacillus subtilis* (*B. subtilis*),* Bacillus stearothermophilus* (*B. stearothermophilus*), and* Bacillus licheniformis* (*B. licheniformis*) are known to be good producers of *α*-amylase and these have been widely used for commercial production of the enzyme for various applications [5]. So far, a number of *α*-amylase genes have been isolated and characterized from* Bacillus* sp., including* B. licheniformis*,* B. stearothermophilus,* and* B. subtilis* [6–8]. In previous studies, a gene encoding *α*-amylase from* B. licheniformis* was cloned and expressed in* E. coli* and* B. subtilis* [9]. However, the low expression level does not allow the recombinant protein to be applied practically and economically in industry. For commercial exploitation of the recombinant *α*-amylase, it is essential to achieve high yield of the protein.

The methylotrophic yeast* P. pastoris* has many advantages as a host for production of recombinant heterologous proteins, such as high cell density, high levels of productivity, ease of genetic manipulation, the ability to perform complex posttranslational modifications, and very low secretion levels of endogenous proteins [10]. To improve heterologous expression of genes, many strategies have been developed in* P. pastoris*. It includes high copy number of heterologous gene, appropriate signal peptide in expression vector, high efficient transcriptional promoters, and optimization of cell cultivation [11–13]. However, these optimization strategies did not universally result in high protein production for every recombinant protein as expected. It has now been shown that the difference of codon usage between the native gene sequence and expression host has significant impact on the expression level of recombinant protein [14, 15]. Therefore, the codon optimization is a promising technique for increasing foreign protein expression level.

In this study, we describe the high-level expression of* B. licheniformis α*-amylase (BlAmy) in* P. pastoris* and this is the first report about high cell density fermentation for production of recombinant* B. licheniformis α*-amylase (rBlAmy) in 5- and 50-L bioreactor. Furthermore, the *α*-amylase gene from* B. licheniformis* (*BlAmy*) was modified and expressed according to its preferred condon usage in* P. pastoris*. To our knowledge, this is also the first report to improve* B. licheniformis α*-amylase (BlAmy) production by codon optimization strategies in* P. pastoris*.

## 2. Materials and Methods

### 2.1. Strains, Plasmids, Reagents, and Media

The* P. pastoris* strain GS115 and the expression vector pPIC9K were purchased from Invitrogen (Carlsbad, CA, USA). The* E. coli* strain Top 10 is routinely conserved in our laboratory. Restriction enzymes, T_4_-DNA ligase, and Pfu DNA polymerase were purchased from Sangon Biotech (Shanghai, China). All other chemicals used were analytical grade reagents unless otherwise stated. Yeast extract peptone dextrose (YPD) medium, buffered glycerol complex (BMGY) medium, and buffered methanol complex (BMMY) medium were prepared according to the manual of* Pichia* Expression Kit (Version F, Invitrogen). Fermentation Basal Salts (BSM) Medium and PTM1 Trace Salts used for fermentation were prepared according to the* Pichia* Fermentation Process Guidelines (Invitrogen).

### 2.2. Codon Optimization and Synthesis of the Gene

The codon usage of* BlAmy* (GenBank M38570) from* B. licheniformis* was analyzed using Graphical Codon Usage Analyser (http://gcua.schoedl.de/) and was optimized by replacing the codons predicted to be less frequently used in* P. pastoris* with the frequently used ones by (http://www.dna20.com/). Signal peptide was analyzed by SignalP 4.0 server (http://www.cbs.dtu.dk/services/SignalP/). The optimized gene (*BlAmy-opt*) was synthesized by Sangon (Shanghai, China).

### 2.3. Vector Construction

The synthetic gene encoding the mature region of *α*-amylase without the predicted signal sequence was digested by EcoRI and NotI and then ligated into pPIC9K, forming pPIC9K-*BlAmy-opt*. The native *α*-amylase gene (*BlAmy*) from* B. licheniformis* was cloned into pPIC9K using primers BlF (5′-CATCGAATTCGCAAATCTTAATGGGACGCTG-3′) and BlR (5′-CATAGCGGCCGCCTATCTTTGAACATAAATTGA-3′), resulting in the recombinant plasmid pPIC9K-*BlAmy. *The recombinant plasmids were checked by DNA sequencing.

### 2.4. Transformation of* P. pastoris* and Screening Transformants

*P. pastoris* GS115 was transformed with 10 *μ*g of SacI-linearized pPIC9K-*BlAmy-opt* and pPIC9K-*BlAmy* vector by electrotransformation, according to Invitrogen's recommendations. Transformants were initially selected by MD medium (1.34% yeast nitrogen base, 4 × 10^−5^% biotin, 2% dextrose) plates and then checked by colony PCR. The insertion copy number of transformants was determined by their resistance to G418 and transformants with the same copy number were selected. The recombinants carrying* BlAmy* and* BlAmy-opt* were screened by a new plate assay method. The recombinants from 1.0, 2.0, and 4.0 mg/mL G418-YPD plate were picked and cultured in 96-deep-well microplates containing 200 *μ*L/well BMGY medium at 30°C for 24 h. After this, the cells were harvested by centrifugation, resuspended, and cultured in 400 *μ*L/well BMMY medium. After 24 h, plates were subjected to centrifugation again and supernatants were used in subsequent activity assays. Qualitative *α*-amylase activity was determined by a halo plate assay containing 3% (w/v) agar and 5% (w/v) soluble starch (Sangon, China). Supernatants (20 *μ*L) were loaded into wells and plates were incubated at 60°C for 30 min. The clones were selected according to the size of the halos and their activities were checked by shaking flask fermentation.

### 2.5. Expression of* BlAmy-opt* and BlAmy in* P. pastoris* Shake-Flask Cultures

The transformants were selected and cultivated in shaking flask. The seeds were inoculated in 10 mL of BMGY medium in a 100 mL shake flask and incubated at 30°C and 250 rpm until the culture reached an OD600 = 2.0–6.0. The cells were harvested by centrifugation and resuspended in 50 mL of BMMY medium and incubated at 30°C and 200 rpm. The methanol induction temperature was set at 30°C, and 0.7% (v/v) methanol was fed at 24 h intervals for 5 days. The activities of the *α*-amylase were checked at 24, 48, 72, 96, 120, 144, and 168 h. The colony with the highest activity was selected as the strain to ferment in 5- and 50-L bioreactor.

### 2.6. High Cell Density Fermentation

The transformed strain showing the highest *α*-amylase activity in shake-flask culture was cultivated in high cell density fermentor. High cell density fermentation was carried out in 5- and 50-L bioreactor (Baoxing Co., Shanghai, China). The cultivation conditions and medium composition was the same as the previous described method [16]. Inoculum was cultured in BMGY medium. Cells were grown for 18–20 h at 30°C on shaker of 200 rpm. Then, 10% (v/v) of the inoculum was inoculated into the 5- and 50-L bioreactors containing 2- and 20-L basal salt medium, made of 0.47 g/L CaSO_4_·2H_2_O, 9.1 g/L K_2_SO_4_, 7.5 g/L MgSO_4_·7H_2_O, 6.2 g/L KOH, 13.35 mL/L H_3_PO_4_ (85%), 20.0 g/L glycerol, and 1.5 mL* Pichia* trace metal 1 (PTM1). One liter PTM1 consists of 6 g CuSO_4_·5H_2_O, 0.08 g NaI, 3 g MnSO_4_·H_2_O, 0.5 g CoCl_2_, 20 g ZnCl_2_, 0.02 g H_3_BO_3_, 0.2 g Na_2_MnO_4_·2H_2_O, 65 g FeSO_4_·7H_2_O, 0.2 g biotin, and 30 mL 6 N H_2_SO_4_. The temperature was controlled at 30°C and the pH was maintained at 5.0 using NH_4_OH (28%) and H_3_PO_4_ (10%). For 5 L bioreactor, the agitation rate was set at 600 rpm and the aeration rate was 30 L/min. For 50 L bioreactor, the agitation rate was set at 500 rpm and the aeration rate was 40 L/min. When glycerol was used up, as indicated by an increase in dissolved oxygen (DO), 0.5% (v/v) methanol was added to induce expression *α*-amylase. Feeding of methanol was linked to the dissolved oxygen (DO). When the initial methanol 0.5% (v/v) was depleted (indicated by an abrupt increase in DO), 100% methanol solution containing 1.2% (v/v) PTM1 was added automatically. The concentration of methanol was kept stable by monitoring the dissolved oxygen (OD) content and maintaining it at greater than 20%. The enzyme activity of the supernatant and dry cell weight were monitored throughout the cultivation.

### 2.7. Purification, Deglycosylation, and SDS-PAGE Analysis of Recombinant BlAmy

After fermentation, cells from the cultures were removed by centrifuging at 6000 ×g for 10 min. The supernatant was concentrated by ultrafiltration using a Millipore set-up according to the manufacturer's instructions with a membrane of 10 kDa cut-off. The supernate containing recombinant BlAmy was purified by 2 mL Ni^2+^-chelating chromatography according to the manuals (Biorad, USA). The elution buffer containing purified recombinant BlAmy was used for further analysis. Purified recombinant BlAmy was deglycosylated using 300 U of Endo H for 3 h at 37°C according to the manufacturer's instructions (NEB, USA). The deglycosylated and untreated were analyzed by SDS-PAGE. SDS-PAGE was carried out on a 12% running gel and stained with Coomassie Blue.

### 2.8. Assay of **α**-Amylase Activity and Protein Determination

*α*-Amylase activity was assayed according to the method described by previous studies [17]. One unit of *α*-amylase was defined as the amount of amylase needed to complete the liquefaction of 1 mg of starch into dextrin per minute at 70°C and pH 6.0. The protein content was determined according to the Bradford method using BSA as standard.

### 2.9. Characterization of the Recombinant BlAmy and Deglycosylated Recombinant BlAmy

The relative enzyme activity was determined at various pH values using 100 mM buffers, pH ranging from 4.0 to 11.0. Buffers used as standard were sodium acetate buffer (pH 4–6), sodium phosphate buffer (pH 6–8), and sodium carbonate buffer (pH 9–11). To evaluate the pH stability, aliquots of enzyme samples were incubated at 30°C for 24 h with respective pH buffers. Remaining enzyme activity was measured under standard assay protocol and calculated considering the initial activity. The optimal temperature of the enzyme was determined by measuring the enzyme activity at various temperatures (40–100°C) in 100 mM of sodium phosphate buffer, pH 7.0. Thermal stability was determined by incubating the purified enzyme in 100 mM of sodium phosphate buffer (pH 7.0) for 1 h at the desired temperatures (60–100°C) followed by measuring the residual activity.

## 3. Results and Discussion

### 3.1. Sequence Optimization and De Novo Synthesis of BlAmy

**Figure 1:**
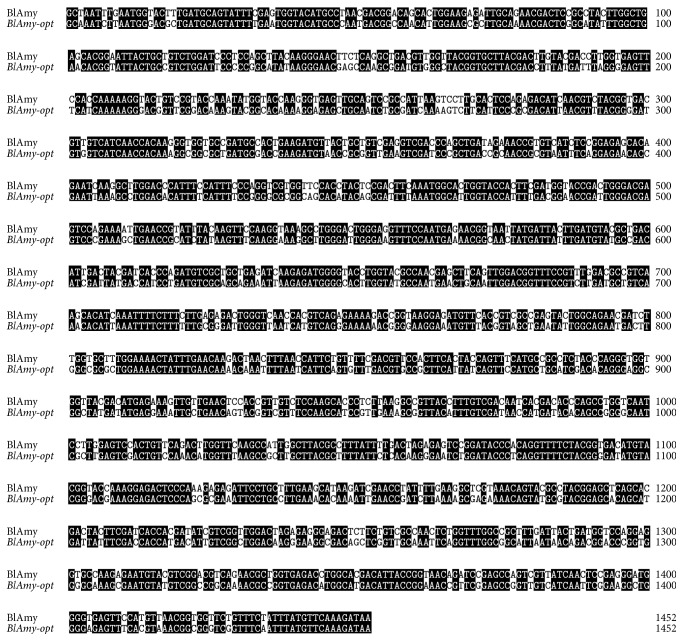
Sequence comparison between the original (*BlAmy*) and the optimized (*BlAmy-opt*) genes. Identical residues are marked in black background.

*P. pastoris* has been routinely used as a heterologous expression system because of its efficient secretion, high expression level, and high cell density [10]. However, the bias of codon usage between the native gene sequence and* P. pastoris* has significant impact on the expression level of recombinant protein. Codon optimization by using frequently used codons in the host is an efficient measure to improve the expression level of heterologous gene. Generally, this is accomplished by replacing all codons with preferred codons, eliminating AT-rich stretches and adjusting the G+C content [18, 19]. Analysis of the DNA sequence of native *α*-amylase gene (BlAmy) using Graphical Codon Usage Analyser revealed that some amino acid residues were encoded by codons that are rarely used in* P. pastoris*, codons like GGC (Gly), GCG (Ala), AGC (Ser), TCG (Ser), and CCG (Pro); most of them are shared less than 15% of usage percentage, which may result in a much lower expression level in* P. pastoris*. In order to achieve a high-level expression of BlAmy in* P. pastoris*, the codons of BlAmy were replaced with those more frequently used by* P. pastoris* (Table 1). The codon adaptation index (CAI) of the native* BlAmy* was improved from 0.74 to 0.86. Furthermore, the G+C content was increased from 47.6 to 49.2%, which was closer to the G+C content of other high-expression genes in* P. pastoris*. The nucleotides A, T, G, and C dispersed evenly in the synthesized gene to eliminate AT- or GC-rich motifs, codons containing both AT and GC were selected when the differences between the codon frequencies were not significant. Totally, the codons encoding 305 amino acids were optimized in which a total of 328 nucleotides were changed (Table 1). The optimized gene (*BlAmy-opt*) shared 77% of nucleotide sequence identity with that of the native gene (*BlAmy*) (Figure 1).

### 3.2. Vector Construction and Selection of Producing Clones

**Figure 2:**
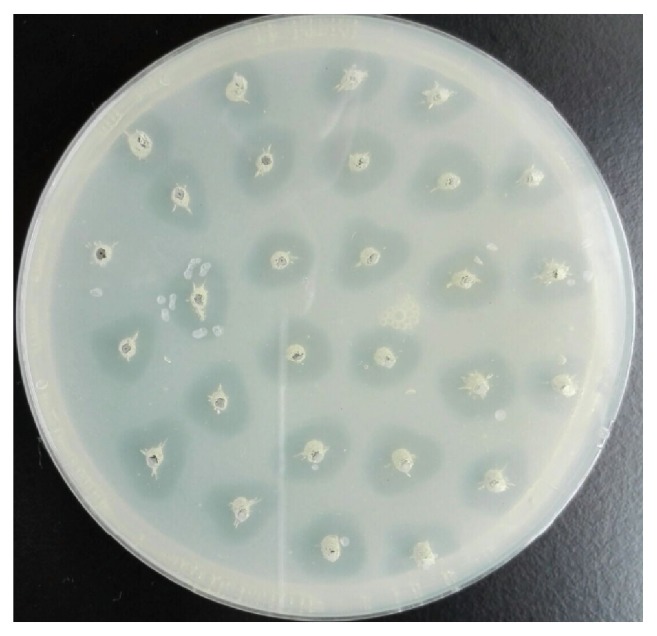
Screening of strains with higher level of *α*-amylase production by plate assay. The agar plates contain 3% (w/v) agar and 5% (w/v) soluble starch.

The recombinant plasmids pPIC9K-*BlAmy-opt* and pPIC9K-*BlAmy* were linearized and transformed into* P. pastoris* GS115 and several thousands of transformants were obtained on MD plates. In this study, the putative multicopy inserts were selected for expression by screening with the same concentration of G418. The positive clones (from 1.0, 2.0, and 4.0 mg/mL G418-YPD plate) were cultured in 96-deep-well microplates and further screened by a new halo plate assay (Figure 2). According to the size of the halos, twenty clones (ten isolated from recombinants carrying* BlAmy-opt* and ten from recombinants carrying* BlAmy*, resp.) from 2.0 mg/mL G418-YPD plate were selected for shake-flask cultures. The plate assay is a simple, rapid, and well adapted method for screening of large number of samples [20]. The diameter of the halo zone is very useful for predicting the enzyme yield as an aid to select strains with a high level of *α*-amylase production.

### 3.3. Expression of* BlAmy-opt* and BlAmy in* P. pastoris* at Shaking Flask Level

Twenty colonies with larger halos were selected and cultivated in shaking flask. In shaking flask, the *α*-amylase activity increased gradually and reached the highest activity after 144 h of cultivation. After 144 h of cultivation under inducing conditions, the *α*-amylase activity of the supernatant from different clones carrying* BlAmy-opt* varied between 310 and 420 U/mL (enhanced 5.25-fold compared with the wild strain* Bacillus licheniformis*), while the recombinants carrying* BlAmy* varied between 150 and 230 U/mL (enhanced 2.87-fold compared with the wild strain* Bacillus licheniformis*), respectively. Two clones (one carrying* BlAmy-opt*, the other carrying* BlAmy*) from YPD plate containing 2.0 mg/mL showed the highest *α*-amylase activity of 420 U/mL and 230 U/mL in shaking flask culture was chosen for high cell density fermentation.

### 3.4. High Cell Density Fermentation

**Figure 3:**
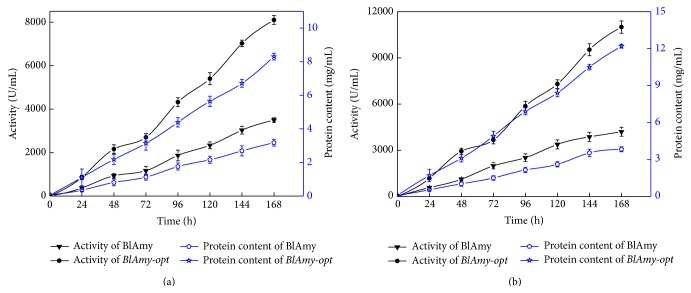
*α*-Amylase activity and total protein content in basal salt medium at 30°C and pH 5.0 during fed-batch fermentation in 5-L (a) and 50-L (b) bioreactor. *α*-Amylase activity was determined by starch-iodine color method; the protein content was determined according to the Bradford method using BSA as standard. All measurements were carried out in triplicate.

**Figure 4:**
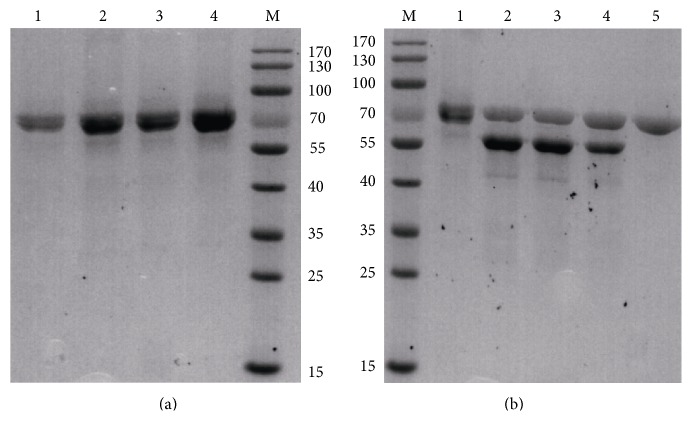
SDS-PAGE analysis of recombinant BlAmy. (a) SDS-PAGE of recombinant BlAmy in fermentation broth from 5- and 50-L bioreactor after methanol induced for 168 h. M: protein MW markers; lane 1 and lane 2: recombinant BlAmy and* BlAmy-opt* from 5-bioreactor, respectively. Lane 3 and lane 4: recombinant BlAmy and* BlAmy-opt* from 50-bioreactor, respectively. (b) Analysis of purified recombinant BlAmy and *N*-deglycosylated recombinant BlAmy by Endo H. Lane 1: purified recombinant BlAmy; lane 2, lane 3, and lane 4: the *N*-deglycosylated recombinant BlAmy and Endo H; lane 5: Endo H.

To obtain a large amount of active protein, fed-batch studies were carried out in 5- and 50-L fermentor. Upon methanol induction, the maximum *α*-amylase activity and protein concentration produced by recombinant strain GS115 carrying* BlAmy-opt* reached 8100 U/mL and 8.3 g/L, respectively, in the 5-L fed batch bioreactor (Figure 3(a)). Compared with the expression of the native gene in* P. pastoris* (3500 U/mL), the expression level of codon optimized gene was increased by 2.31-fold (Figure 3(a)). The maximum *α*-amylase activity and protein concentration of recombinant strain GS115 carrying* BlAmy-opt* obtained in the 50-L fed-batch bioreactor were 11000 U/mL and 12.2 g/L, respectively (Figure 3(b)). Compared with the expression of the native gene in* P. pastoris* (4200 U/mL), the expression level of codon optimized gene was increased by 2.62-fold (Figure 3(b)). The recombinant protein accounted for 86% of the total protein in the medium as estimated by the Software Quantity One (Figure 4(a)).

As an easy and simple system,* P. pastoris* is now widely used for heterologous production of recombinant proteins [21]. Due to the difference of codon usage between the native gene sequence and expression host, researchers have used codon optimization to increase the expression level of heterologous genes in* P. pastoris*. By codon optimization, the expression of xylanase gene from* Thermotoga maritime* and* Aspergillus sulphureus* was improved 2.8- and 5-fold, respectively [18, 22]. The optimization of glucanase gene from* B. licheniformis* and* Fibrobacter succinogenes* resulted in a 10- and 2.34-fold increase of target protein production [15, 23]. In this study, the* BlAmy-opt* was expressed in* P. pastoris* at a significantly higher level (12.2 g/L) with *α*-amylase activity of 11000 U/mL in 50-L fermentor after 168 h induction through codon optimization. These results showed that codon optimization is an effective method to increase the expression of foreign protein in* P. pastoris*. Meanwhile, the codon optimized recombinant *α*-amylase has a great potential use in industrial application due to its high-expression level.

### 3.5. SDS-PAGE Analysis of Recombinant BlAmy

As shown in Figure 4(a), the purified recombinant BlAmy showed two forms of BlAmy with molecular masses close to 70 kDa, which is about 11 kDa larger than 58.5 kDa, the calculated molecular weight of the nonglycosylated BlAmy. As shown in Figure 4(b), Endo H treatment of recombinant BlAmy resulted in a shift in the protein band on SDS-PAGE and yielded a single band of 58 kDa, suggesting that the two forms of recombinant BlAmy contained different degree of glycosylation.

### 3.6. Characterization of the Recombinant BlAmy and Deglycosylated Recombinant BlAmy

**Figure 5:**
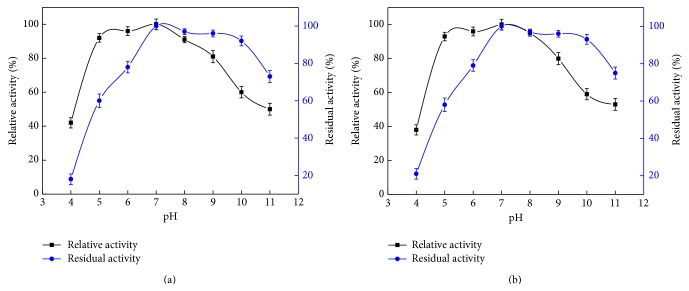
Influence of pH on activity and stability of recombinant BlAmy (a) and deglycosylated recombinant BlAmy (b). *α*-Amylase activity was determined by starch-iodine color method. Optimal pH was determined by assessing the activity of the purified recombinant BlAmy at pH 4.0–11.0. The relative activity at different pH values was calculated by setting pH 7.0 as 100%. The pH stability was determined by measuring the residual enzyme activities after incubating purified recombinant BlAmy at various pH for 24 h at 30°C. The residual activity was calculated by taking the activity of purified recombinant BlAmy without buffer treatment as 100%. All measurements were carried out in triplicate.

The influence of pH on recombinant* BlAmy-opt* and deglycosylated recombinant BlAmy activity and stability are presented in Figure 5. The activity of recombinant* BlAmy-opt* and deglycosylated recombinant BlAmy were measured over a pH range of 4.0–11.0. As shown in Figure 5(a), the recombinant* BlAmy-opt* remained active at a pH range of 5.0–9.0 and showed maximum activity at pH 7.0, which is similar to the deglycosylated recombinant BlAmy (Figure 5(b)). In the pH stability study, the recombinant* BlAmy-opt* and deglycosylated recombinant BlAmy are stable at abroad range of pH values between pH 6.0 and 10.0 after 24 h incubation at 30°C, retaining over 78% of its initial activity.

**Figure 6:**
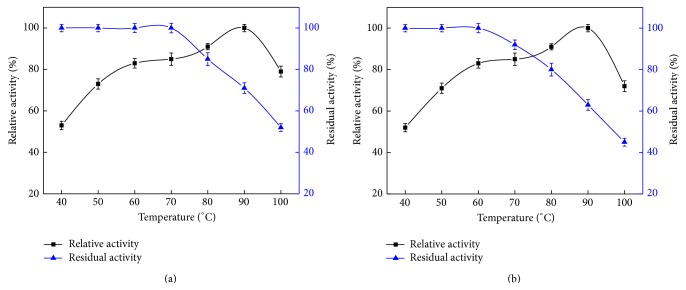
Influence of temperature on activity and stability of recombinant BlAmy (a) and deglycosylated recombinant BlAmy (b). *α*-Amylase activity was determined by starch-iodine color method. The optimum temperature of purified recombinant BlAmy was measured at different temperatures ranging from 40 to 100°C. The relative activity at different temperatures was calculated by setting 90°C as 100%. The thermal stability was studied by incubating lipase at various temperatures (40–100°C) in sodium phosphate buffer (pH 7.0) up to 1 h. The residual enzyme activity was measured at 70°C and the residual activity was calculated by taking the nonheated lipase activity as 100%. All measurements were carried out in triplicate.

The activity of recombinant* BlAmy-opt* and deglycosylated recombinant BlAmy were also determined at different temperatures. As shown in Figures 6(a) and 6(b), the recombinant* BlAmy-opt* and deglycosylated recombinant BlAmy showed an optimum activity at 90°C and activity dropped above 100°C. Thermostability was examined by incubating the recombinant* BlAmy-opt* and deglycosylated recombinant BlAmy at different temperatures for 1 h, and the residual activity was measured at 70°C under the conditions mentioned above. The activity of the recombinant* BlAmy-opt* was almost not affected by a temperature below 70°C, but it decreased dramatically when the temperature was above 90°C. The thermostability of BlAmy was higher than deglycosylated recombinant BlAmy. BlAmy showed 71 and 53% residual activity after 1 h incubation at 90 and 100°C, whereas deglycosylated recombinant BlAmy showed only 62 and 43%.

## 4. Conclusions

In this study we report the high-level expression of BlAmy in* P. pastoris*. The results showed that* P. pastoris* is an excellent host to production of BlAmy. To our knowledge, this is the first report about high cell density fermentation for production of recombinant BlAmy in 5- and 50-L bioreactor. Meanwhile, we developed a new simple and quick plate assay for screening of strains with higher level of *α*-amylase production. The most striking success in this study was that we improved the expression of BlAmy in* P. pastoris* by rewriting native* BlAmy* according to* P. pastoris* preferred codon usage. The results showed that codon optimization is an effective method to increase the expression of foreign protein in* P. pastoris*. Meanwhile, the results presented here will greatly contribute to improving production of recombinant BlAmy and offer a greater value in various industrial applications.

## Figures and Tables

**Table 1:** Comparison of the codon usage for wild-type and synthetic *α*-amylase gene targeted at *P. pastoris* for expression.

AA	Codon	Host fraction	*BlAmy *	*BlAmy*-*opt *
Gly	GGG	0.10	11	0
	GGA	0.32	12	11
	GGT	0.44	6	34
	GGC	0.14	16	0

Glu	GAG	0.43	7	21
	GAA	0.57	18	4

Asp	GAT	0.58	19	11
	GAC	0.42	18	26

Val	GTG	0.19	4	0
	GTA	0.15	4	0
	GTT	0.42	12	14
	GTC	0.23	12	18

Ala	GCG	0.06	9	0
	GCA	0.23	10	0
	GCT	0.45	10	19
	GCC	0.26	7	17

Arg	AGG	0.16	4	0
	AGA	0.48	3	15
	CGG	0.05	3	0
	CGA	0.10	3	0
	CGT	0.16	2	7
	CGC	0.05	7	0

Lys	AAG	0.53	11	20
	AAA	0.47	17	8

Ser	AGT	0.15	2	0
	AGC	0.09	4	0
	TCG	0.09	8	0
	TCA	0.19	4	0
	TCT	0.29	3	9
	TCC	0.20	5	17

Stop	TAA	0.53	1	1

Asn	AAT	0.49	11	5
	AAC	0.51	14	20

Met	ATG	1.00	7	7

Ile	ATA	0.19	1	0
	ATT	0.50	13	10
	ATC	0.30	6	10

Thr	ACG	0.11	7	0
	ACA	0.24	13	0
	ACT	0.40	3	12
	ACC	0.25	4	15

Trp	TGG	1.00	17	17

Cys	TGT	0.65	0	0
	TGC	0.35	0	0

Tyr	TAT	0.46	18	5
	TAC	0.55	12	25

Leu	TTG	0.33	11	23
	TTA	0.16	3	0
	CTG	0.16	6	0
	CTA	0.11	0	0
	CTT	0.16	7	5
	CTC	0.08	1	0

Phe	TTT	0.54	16	5
	TTC	0.46	4	15

Gln	CAG	0.39	9	15
	CAA	0.61	11	5

His	CAT	0.57	17	6
	CAC	0.43	7	18

Pro	CCC	0.15	3	0
	CCG	0.09	8	0
	CCA	0.41	1	9
	CCT	0.35	3	6

## References

[1] Gupta R., Gigras P., Mohapatra H., Goswami V. K., Chauhan B. (2003). Microbial *α*-amylases: a biotechnological perspective. *Process Biochemistry*.

[2] Janeček Š., Svensson B., MacGregor E. A. (2013). *α*-Amylase: an enzyme specificity found in various families of glycoside hydrolases. *Cellular and Molecular Life Sciences*.

[3] Crabb W. D., Mitchinson C. (1997). Enzymes involved in the processing of starch to sugars. *Trends in Biotechnology*.

[4] Sivaramakrishnan S., Gangadharan D., Nampoothiri K. M., Soccol C. R., Pandey A. (2006). *α*-amylases from microbial sources—an overview on recent developments. *Food Technology and Biotechnology*.

[5] de Souza P. M., Magalhães P. O. (2010). Application of microbial *α*-amylase in industry—a review. *Brazilian Journal of Microbiology*.

[6] Liu H., Yang J. K., Yan Y. J. (2007). Cloning and expression of an *α*-amylase gene from *Bacillus subtilis*. *Biotechnology*.

[7] Ren D. M., He C. G., Qian Z. W., Deng X. C., Yang Q. Y. (1987). Cloning and expression of two *α*-amylase gene from *Bacillus stearothermophilus* in *Ecoli*. *Chinese Journal of Biotechnology*.

[8] Yuuki T., Nomura T., Tezuka H. (1985). Complete nucleotide sequence of a gene coding for heat- and pH-stable *α*-amylase of Bacillus licheniformis: comparison of the amino acid sequences of three bacterial liquefying *α*-amylases deduced from the DNA sequences. *The Journal of Biochemistry*.

[9] Joyet P., Guerineau M., Heslot H. (1984). Cloning of a thermostable *α*-amylase gene from *Bacillus licheniformis* and its expression in *Escherichia coli* and *Bacillus subtilis*. *FEMS Microbiology Letters*.

[10] Macauley-Patrick S., Fazenda M. L., McNeil B., Harvey L. M. (2005). Heterologous protein production using the *Pichia pastoris* expression system. *Yeast*.

[11] Ramón R., Ferrer P., Valero F. (2007). Sorbitol co-feeding reduces metabolic burden caused by the overexpression of a Rhizopus oryzae lipase in *Pichia pastoris*. *Journal of Biotechnology*.

[12] Yu M., Wen S., Tan T. (2010). Enhancing production of *Yarrowia lipolytica* lipase Lip2 in *Pichia pastoris*. *Engineering in Life Sciences*.

[13] Wang X.-F., Shen X.-G., Sun Y.-C. (2012). Production of *Yarrowia lipolytica* lipase LIP2 in *Pichia pastoris* using the nitrogen source-regulated *FLD1* promoter. *Journal of Chemical Technology and Biotechnology*.

[14] Xiong A.-S., Yao Q.-H., Peng R.-H. (2006). High level expression of a synthetic gene encoding *Peniophora lycii* phytase in methylotrophic yeast *Pichia pastoris*. *Applied Microbiology and Biotechnology*.

[15] Teng D., Fan Y., Yang Y.-L., Tian Z.-G., Luo J., Wang J.-H. (2007). Codon optimization of *Bacillus licheniformisβ*-1,3-1,4-glucanase gene and its expression in *Pichia pastoris*. *Applied Microbiology and Biotechnology*.

[16] Wang J.-R., Li Y.-Y., Xu S.-D., Li P., Liu J.-S., Liu D.-N. (2013). High-level expression of pro-form lipase from *Rhizopus oryzae* in *Pichia pastoris* and its purification and characterization. *International Journal of Molecular Sciences*.

[17] Liu Y., Fan S., Liu X. (2014). A highly active alpha amylase from *Bacillus licheniformis*: directed evolution, enzyme characterization and structural analysis. *Journal of M icrobiology and Biotechnology*.

[18] Jia H. Y., Fan G. S., Yan Q. J., Liu Y. C., Yan Y., Jiang Z. Q. (2012). High-level expression of a hyperthermostable *Thermotoga maritima* xylanase in *Pichia pastoris* by codon optimization. *Journal of Molecular Catalysis B: Enzymatic*.

[19] Yang J., Liu L. (2010). Codon optimization through a two-step gene synthesis leads to a high-level expression of *Aspergillus niger lip2* gene in *Pichia pastoris*. *Journal of Molecular Catalysis B: Enzymatic*.

[20] Ten L. N., Im W.-T., Kim M.-K., Kang M. S., Lee S.-T. (2004). Development of a plate technique for screening of polysaccharide-degrading microorganisms by using a mixture of insoluble chromogenic substrates. *Journal of Microbiological Methods*.

[21] Lan D.-M., Yang N., Wang W.-K., Shen Y.-F., Yang B., Wang Y.-H. (2011). A novel cold-active lipase from *Candida albicans*: cloning, expression and characterization of the recombinant enzyme. *International Journal of Molecular Sciences*.

[22] Li Y., Zhang B., Chen X., Chen Y., Cao Y. (2010). Improvement of *Aspergillus sulphureus* endo-*β*-1,4-xylanase expression in *Pichia pastoris* by codon optimization and analysis of the enzymic characterization. *Applied Biochemistry and Biotechnology*.

[23] Huang H., Yang P., Luo H. (2008). High-level expression of a truncated 1,3-1,4-*β*-D-glucanase from *Fibrobacter succinogenes* in *Pichia pastoris* by optimization of codons and fermentation. *Applied Microbiology and Biotechnology*.

